# Supplementary material for: Patient information needs for transparent and trustworthy cardiovascular artificial intelligence: A qualitative study
Source: PLOS Digit Health. 2025 Apr 21;4(4):e0000826. doi: 10.1371/journal.pdig.0000826 (PMC12011294; doi:10.1371/journal.pdig.0000826)
Supplement: S1 Table — Adapted from [32]. (DOCX) [file pdig.0000826.s001.docx]

**S1 Table: Standards for Reporting Qualitative Research (SRQR)**

| **No.** | **Topic** | **Page(s) Reported** |
| --- | --- | --- |
|  | **Title and abstract** |  |
| S1 | Title | 1 |
| S2 | Abstract | 2 |
|  | **Introduction** |  |
| S3 | Problem formulation | 4-5 |
| S4 | Purpose or research question | 6 |
|  | **Methods** |  |
| S5 | Qualitative approach and research paradigm | 6-7 |
| S6 | Researcher characteristics and reflexivity | 7 |
| S7 | Context | 6-7 |
| S8 | Sampling strategy | 7-8 |
| S9 | Ethical issues pertaining to human subjects | 7 |
| S10 | Data collection methods | 8-10 |
| S11 | Data collection instruments and technologies | 8-10 |
| S12 | Units of study | 11 |
| S13 | Data processing | 10 |
| S14 | Data analysis | 10 |
| S15 | Techniques to enhance trustworthiness | 9-10 |
|  | **Results/findings** |  |
| S16 | Synthesis and interpretation | 11, 13 |
| S17 | Links to empirical data | 11-20 |
|  | **Discussion** |  |
| S18 | Integration with prior work, implications, transferability, and contribution(s) to the field | 20-24 |
| S19 | Limitations | 25-26 |
|  | **Other** |  |
| S20 | Conflicts of interest | N/A - journal competing interest form |
| S21 | Funding | N/A - financial disclosure form |

*Adapted from*: O'Brien BC, Harris IB, Beckman TJ, Reed DA, Cook DA. Standards for reporting qualitative research: a synthesis of recommendations. *Acad Med*. 2014;89(9):1245-51. doi: 10.1097/ACM.0000000000000388. PubMed PMID: 24979285.
