# Supplementary material for: Patient information needs for transparent and trustworthy cardiovascular artificial intelligence: A qualitative study
Source: PLOS Digit Health. 2025 Apr 21;4(4):e0000826. doi: 10.1371/journal.pdig.0000826 (PMC12011294; doi:10.1371/journal.pdig.0000826)
Supplement: S2 Table — (DOCX) [file pdig.0000826.s002.docx]

**S2 Table: Supplemental Quotations for Information Factors**

| **Information factor** | **Focus Group** | **Quotation** |
| --- | --- | --- |
| ***Information about the AI tool*** |  |  |
| Name | FG2 | Name of the software, maybe where it was created like the name of the research lab or university it was created in […] |
| Manufacturer | FG2 | Provide [the] name of the entity that created it. |
| Purpose/functionality | FG3 | Exactly how it [AI] works, what it does and what human processes it eliminates. |
| Patient data used/accessed | FG1 | What data is collected and how it is intended to be used? |
| Performance | FG1 | I would want to know the accuracy of the intelligence--what is the rate of false positives. |
| Accuracy | FG3 | I would want to know how accurate the results are. |
| Reliability | FG1 | I would want to know about the reliability of the AI software. |
| Effectiveness | FG2 | I would want to know if this was effective in addressing my health issues. |
| Generalizability | FG3 | Is AI capable of adapting to treating me the individual versus a patient type. |
| Diverse data sets | FG3 | I would hope that AI testing/research is done with diverse populations unlike what currently occurs in healthcare research. |
| Diverse intended populations | FG2 | I'd want to know about the demographics of the people […] I've read about research, indicating that people of different racial backgrounds respond differently to various medications and treatments. |
| Limitations | FG1 | […] caveats (limitations on accuracy and interpreting the results) are necessary. |
| Risks | FG2 | I like the idea of including information on possible risks. It’s important to be informed about those. |
| ***Information about oversight*** |  |  |
| Certifications/endorsements | FG3 | Trusting the AI results or recommendations to me is built on effectiveness and honest reviews gathered from random people. |
| Regulatory approval | FG2 | As long as the AI tool was FDA approved, I would be okay with it making the decision. |
| Physician endorsement | FG3 | I would prefer my doctor to recommend the best AI software to use and just inform me on how it works. |
| Patient endorsement | FG3 | I need to see […] reviews from other patients who have make [sic] use of this software before. |
| Conflicts of interest | FG1 | […] if there are any conflicts of interest. |
| Data security | FG2 | How is my data being used, protected, and shared across systems? |
| Human oversight | FG1 | I'd want to know how often / how much a human medical professional is looking at the data for accuracy. |
| Care team training/experience | FG1 | How safe is it? If my care team knows what they are doing and uses the equipment/technology completely and correctly. |
| Verification of outputs | FG3 | I could trust the AI results and recommendations if they were reviewed and confirmed by a doctor. |
| ***Impact on care experience*** |  |  |
| Use in clinical decision-making | FG1 | I would very much like to know its application and use in my care […] I would like to discuss with my provider the intent of its use and how its recommendations are used by the team. |
| Added value/efficiency | FG2 | I would want to know what the benefit of it is, are they using it to better my care or make it easier on doctors/PCP? |
| Comparison to standard of care | FG3 | I would want to know how it [AI ECG] compares to traditional ecg tracing. |
| Financial costs | FG2 | I would want to know how much this is costing me. |
