# Supplementary material for: Patient information needs for transparent and trustworthy cardiovascular artificial intelligence: A qualitative study
Source: PLOS Digit Health. 2025 Apr 21;4(4):e0000826. doi: 10.1371/journal.pdig.0000826 (PMC12011294; doi:10.1371/journal.pdig.0000826)
Supplement: S1 Appendix — (DOCX) [file pdig.0000826.s003.docx]

**S1 Appendix: Example Clinical Vignette**

A patient comes to the emergency department with chest pain and shortness of breath. Dr. Rodriguez, an emergency medicine intern in training, takes a full history and performs a physical exam of the patient’s heart and lungs. During her examination, Dr. Rodriguez uses an artificial intelligence enabled stethoscope that provides enhanced listening of the patient’s heart and creates a recording for the physician to review after the examination. The artificial intelligence can detect nuances in heart sounds that may go unnoticed by inexperienced ears. After completing her examination, Dr. Rodriguez identifies a preliminary diagnosis of a heart murmur, sees the stethoscope’s readings also indicate that a heart murmur is likely, and schedules cardiology appointment for the patient to follow-up.
