# Supplementary material for: Patient information needs for transparent and trustworthy cardiovascular artificial intelligence: A qualitative study
Source: PLOS Digit Health. 2025 Apr 21;4(4):e0000826. doi: 10.1371/journal.pdig.0000826 (PMC12011294; doi:10.1371/journal.pdig.0000826)
Supplement: Attachment — Submitted filename: Comments.docx [file pdig.0000826.s004.docx]

1. Lack of Actionable Recommendations: While the findings highlight patient-centered engagement, the text does not provide specific strategies or recommendations for implementing these insights in clinical practice.
2. Complex Sentence Structure: The introduction features long and convoluted sentences, which can obscure the main points. For instance, the sentence discussing "calibrated trust" could be simplified for better understanding. Some sentences are lengthy and complex, which can hinder readability. Breaking them into shorter, more concise statements would improve clarity.
3. Repetition of Concepts: Some ideas, such as the importance of transparency for trust, are reiterated without adding new insights. Streamlining these points could improve conciseness.
4. Lack of Context for Previous Research: The introduction references various studies but does not provide enough context about their findings or relevance to the current study.
5. Potential Biases in Recruitment: The recruitment strategy mentions using ResearchMatch but does not address potential biases in participant selection or limitations of this approach. Acknowledging these issues would provide a more balanced view.
6. Recommendations: Elaborate on Vignette Selection: Offer a more thorough explanation of why the specific vignettes were chosen and how they cover different aspects of healthcare AI.
