# Supplementary material for: Patient information needs for transparent and trustworthy cardiovascular artificial intelligence: A qualitative study
Source: PLOS Digit Health. 2025 Apr 21;4(4):e0000826. doi: 10.1371/journal.pdig.0000826 (PMC12011294; doi:10.1371/journal.pdig.0000826)
Supplement: Attachment — Submitted filename: Review Comments on PDIG.docx [file pdig.0000826.s005.docx]

**Review Comments on PDIG-D-24-00340**

Thank you for inviting me to review this paper. This has been informative and educative for me. The manuscript generally offers new insights and new data, and it is clear that this area is under-research. I believe this paper will offer the required insights for others to follow suit. I must also add that the subject matter area (Artificial Intelligence) is not one that I am very familiar with but has limited knowledge in the area. This may affect my comment on this manuscript to an extent.

**Background**

That being said, I am confident that the manuscript has a very good rigor to it. I was particularly interested in questioning the methodology (being that the qualitative approach is my research strength) but found that every decision was justified scientifically. However, my concern regarding the methodology is the richness of the responses provided by the study's virtual participants. I am saying this because ***speaking*** has been established to communicate feelings, attitudes, and perspectives more than informal writing. Personally, I also believe that people tend to exhibit laziness in responding to questions through writing (as long as they are not taking examinations).

**Method Section**

While I agree that we can generate quantitative data in a qualitative paper, the authors still need to emphasize how or why they had to include multiple-choice questions. This, I believe was not clear in the paper.

**Result Section**

Please shrink the excerpts to 1.0 line spacing in place of what is currently used. It helps to clearly distinguish the excerpts and the manuscript’s texts.

In several sub-themes, you have only included one excerpt. This, I believe may be limiting for your data (particularly because your participants are across the country). What validates the quality of qualitative research is identifying patterns in the participants’ responses, which is not reflected in some components of your study result. In other words, I find it more plausible if you can have a supplementary file where more excerpts per theme and/or sub-themes are included

**Limitation Section**

Kindly provide a heading for the Limitation section

I agree with all the limitations called out.

**Conclusion**

This reads well for me
