# Supplementary material for: Patient information needs for transparent and trustworthy cardiovascular artificial intelligence: A qualitative study
Source: PLOS Digit Health. 2025 Apr 21;4(4):e0000826. doi: 10.1371/journal.pdig.0000826 (PMC12011294; doi:10.1371/journal.pdig.0000826)
Supplement: Attachment — Submitted filename: Peer_Review_Response_PLOSDH_1_21_25.docx [file pdig.0000826.s007.docx]

The authors would like to thank the editor(s) involved for overseeing the review process of our manuscript. We greatly appreciate their time and effort coordinating this process and providing valuable feedback.

In response to the reviewers' and editors' comments, we have thoroughly revised the manuscript. As part of this revised submission, we have included:

1. A revised copy of the manuscript
2. A version with all changes clearly marked
3. A revised supporting information file (S2) along with new files (S1 and S3)

Additional information is available upon request. In accordance with the journal’s data availability policy, the underlying data will be made available on the Open Science Framework (https://osf.io/) upon publication, pending acceptance.

We hope the revisions address all concerns and meet the journal’s standards.

Reviewer #1: Dear Authors,

I want to thank you for the opportunity to review your manuscript entitled "Patient information needs for transparent and trustworthy artificial intelligence in healthcare: A qualitative study." Your work is a valuable contribution to the growing body of literature addressing the intersection of artificial intelligence and healthcare, offering a novel exploration of patient perspectives.

While I commend the novelty of your study, I would like to offer some suggestions for improvement that further enhance your manuscript's clarity and methodological rigor.

RESPONSE: We would like to express our sincere gratitude to Reviewer #1 for the time and careful consideration given to reviewing the manuscript. Their detailed feedback and constructive comments have been invaluable in improving the quality of the work.

1. Title suggestion: given that your research is conducted within the context of cardiovascular healthcare, I recommend reflecting this specific context in the title to make it more precise and to attract a more targeted readership.

RESONSE: We have revised the title to call attention to cardiovascular healthcare.

2. research Design section: the design paragraph would benefit from revision, particularly to avoid redundancy in the description of the data collection strategy. This section is better suited to discussing the epistemological framework underpinning your study, which is constructivism. Clearly articulating this constructivist perspective will help anchor the study's methodology, enhancing transparency for readers.

RESPONSE: We appreciate this suggestion to more clearly articulate the epistemological framework underpinning the study. The social construction of knowledge was indeed a key consideration in our research design. Our revised submission removes redundancy with other parts of the methods and focuses on this underlying framework as well as justifications for our choice of method in alignment with a later comment and comment from Reviewer #4.

3. reporting in general: I recommend adhering to established guidelines for reporting qualitative research, such as the "Consolidated Criteria for Reporting Qualitative Research" (COREQ) or the Standards for Reporting Qualitative Research (SRQR). These guidelines will strengthen the rigor of your study and ensure consistency in reporting.

RESPONSE: Our revised submission includes edits throughout that correspond to standards in the SRQR as well as a supplemental file reporting page number(s) associated with each item. We feel this suggestion has greatly supported our reporting approach and thank Reviewer #1 for this suggestion.

4. Data collection (or as I would like you to say: Data generation or data construction): I encourage you to explain why you chose asynchronous focus groups. While this innovative method is needed, a stronger justification is required to clarify why this approach was deemed suitable for your specific research questions and context. Please also consider to better explain data generation which includes quantitative and qualitative data. This methodological paper may be useful: Braun, V., Clarke, V., Boulton, E., Davey, L., & McEvoy, C. (2020). The online survey as a qualitative research tool. International Journal of Social Research Methodology, 24(6), 641–654. <https://doi.org/10.1080/13645579.2020.1805550>

RESPONSE: We agree with Reviewer #1 that justification for asynchronous online focus groups was lacking in our original submission. Our revised manuscript provides justification for this approach including explanations of several key reasons for selecting this approach along with relevant references. Additionally, we have strengthened our rationale for including quantitative survey data alongside our qualitative findings. We also appreciate the helpful citation provided by Reviewer #1, which has been integrated into our revised methods.

5. Data Analysis: the data analysis approach is somewhat unclear. It is essential to specify whether your analysis followed an inductive, deductive, or combined approach and why you chose to use focus group questions as initial categories for analysis. Additionally, the interpretative contribution of the authors needs to be revised. I suggest reconsidering the findings' narrative to reflect a more interpretative stance to align with a constructivist approach. Please just simply listing themes without accompanying interpretation, as this limits the depth of your analysis.

RESPONSE: We have revised our manuscript to better explain the combination of deductive and inductive approaches we used (deductive for the directed content analysis of the focus group transcripts and inductive analysis to generate a thematic map of information factors).

We also greatly appreciate Reviewer #1’s comments on interpretation. While our analysis follows constructivist principles, we intentionally separated descriptive results from deeper interpretation to maintain clarity. The themes in the Results section lay groundwork for interpretative analysis in the Discussion, where we explore theoretical implications and themes. Our revised Results includes refinement of narrative presentation in some areas to support an appropriate balance between description and interpretation.

6. Rigor: I suggest adding a section on rigor in your methodology, addressing the steps you took to ensure trustworthiness and credibility, such as triangulation, member checking, or reflexivity.

RESPONSE: Our revised manuscript includes additions that focus on improving the presentation of the study’s rigor. While we do not include an explicit subsection devoted to this point, we hope additions throughout our methods provide more insight into the rigor of the study.

7. Method and findings Overlap: please ensure that findings are not inserted into the methods section. This conflation detracts from the clarity of the research process and can need to be clarified regarding the distinction between data collection and interpretation.

RESPONSE: In alignment with a prior comment (comment 5) we have revised our analysis sub-section to clarify our use of deductive and inductive approaches. Our intention was to describe *a priori* categories derived from focus group questions that guided our analysis and not insert findings into our methods. We hope that the revised submission more clearly presents that analysis process and its distinctness from the presented findings.

8. In your limitations, avoid the conventional statement about the lack of generalizability in qualitative research. If generalizability is discussed, I recommend framing it positively by emphasizing the idiographic nature of qualitative research, which is focused on depth and contextual understanding rather than statistical generalization.

RESPONSE: We thank reviewer #1 for providing this framing of qualitative research methods. Our revised manuscript incorporates this recommendation focusing on both depth and authenticity.

Thank you for considering these suggestions, and I look forward to seeing the revised manuscript.

Reviewer #2: 1. Lack of Actionable Recommendations: While the findings highlight patient-centered engagement, the text does not provide specific strategies or recommendations for implementing these insights in clinical practice.

RESPONSE: We agree with Reviewer #2 that specific recommendations are a limitation of our findings. In our revised submission, we have added preliminary considerations for stakeholders while avoiding formal recommendations and reframed our findings as foundational research that enables future work on specific recommendations. This better reflects our study's exploratory aim of identifying key areas for further investigation.

2. Complex Sentence Structure: The introduction features long and convoluted sentences, which can obscure the main points. For instance, the sentence discussing "calibrated trust" could be simplified for better understanding. Some sentences are lengthy and complex, which can hinder readability. Breaking them into shorter, more concise statements would improve clarity.

RESPONSE: The authors have reviewed the full manuscript and have attempted simplify sentences where applicable in the revised submission.

3. Repetition of Concepts: Some ideas, such as the importance of transparency for trust, are reiterated without adding new insights. Streamlining these points could improve conciseness.

RESPONSE: The authors have reviewed the manuscript with an eye toward conciseness and have cut content that may have been repetitive.

4. Lack of Context for Previous Research: The introduction references various studies but does not provide enough context about their findings or relevance to the current study.

RESPONSE: Our revised submission includes a substantially revised introduction that includes a more focused selection of references that are relevant to the present study. We include a more thorough presentation of their findings and relevance as well as greater contextualization of our study in both the Introduction and Discussion.

5. Potential Biases in Recruitment: The recruitment strategy mentions using ResearchMatch but does not address potential biases in participant selection or limitations of this approach. Acknowledging these issues would provide a more balanced view.

RESPONSE: While we noted some limitations regarding our use of ResearchMatch in the initial submission such as the likelihood that participants possessed greater health and technological literacy, our revised manuscript includes deeper reflection of these points and their intersection with potential biases.

6. Recommendations: Elaborate on Vignette Selection: Offer a more thorough explanation of why the specific vignettes were chosen and how they cover different aspects of healthcare AI.

RESPONSE: Our revised manuscript now dedicates a sub-section titled Clinical Vignettes to the explanation of how the vignettes were created and revised for use in the study. We hope this offers more clarity on their selection for our focus groups.

Reviewer #3: The background and relevance have been clearly established.
The study design including the recruitment criteria are adequately explained. The results and discussion are insightful and aligning with the study's objectives.

RESPONSE: We appreciate Reviewer #3’s feedback on the manuscript and thank them for taking the time and effort to review the submission.

Reviewer #4: Thank you for inviting me to review this paper. This has been informative and educative for me. The manuscript generally offers new insights and new data, and it is clear that this area is under-research. I believe this paper will offer the required insights for others to follow suit. I must also add that the subject matter area (Artificial Intelligence) is not one that I am very familiar with but has limited knowledge in the area. This may affect my comment on this manuscript to an extent.

RESPONSE: We thank Reviewer #4 for taking the time to review our manuscript. We particularly appreciate their candid acknowledgment regarding familiarity with AI, as this supported our revisions to ensure the submission remains accessible to readers from various backgrounds.

Background
That being said, I am confident that the manuscript has a very good rigor to it. I was particularly interested in questioning the methodology (being that the qualitative approach is my research strength) but found that every decision was justified scientifically. However, my concern regarding the methodology is the richness of the responses provided by the study's virtual participants. I am saying this because speaking has been established to communicate feelings, attitudes, and perspectives more than informal writing. Personally, I also believe that people tend to exhibit laziness in responding to questions through writing (as long as they are not taking examinations~~).~~

RESPONSE: We agree that there are drawbacks to asynchronous online focus groups. While we address key limitations like moderation challenges and potential misunderstandings in our Limitations section, we have also strengthened our justification for this method in response to Reviewer #1 (comment 4). Our revised submission includes references to literature evaluating the richness of data and thematic content that asynchronous online focus groups can provide.

Method Section
While I agree that we can generate quantitative data in a qualitative paper, the authors still need to emphasize how or why they had to include multiple-choice questions. This, I believe was not clear in the paper.

RESPONSE: In accordance with similar feedback received from Reviewer #1 (comment 4), our revised submission includes additional justification for including multiple-choice questions and their relevance in combination with the qualitative data.

Result Section
Please shrink the excerpts to 1.0 line spacing in place of what is currently used. It helps to clearly distinguish the excerpts and the manuscript’s texts.

RESPONSE: We have reduced the line spacing for quotations.

In several sub-themes, you have only included one excerpt. This, I believe may be limiting for your data (particularly because your participants are across the country). What validates the quality of qualitative research is identifying patterns in the participants’ responses, which is not reflected in some components of your study result. In other words, I find it more plausible if you can have a supplementary file where more excerpts per theme and/or sub-themes are included

RESPONSE: We thank Reviewer #4 for this thoughtful feedback on the presentation of our qualitative data. We have incorporated Reviewer #4’s suggestion and have included a supplemental table that identifies each information factor along with a corresponding participant quotation. We hope this provides additional support for the thematic without overburdening the narrative flow of the manuscript.

Limitation Section
Kindly provide a heading for the Limitation section
I agree with all the limitations called out.

RESPONSE: Our revised submission includes a Limitations heading.

Conclusion
This reads well for me
