# Supplementary material for: Patient information needs for transparent and trustworthy cardiovascular artificial intelligence: A qualitative study
Source: PLOS Digit Health. 2025 Apr 21;4(4):e0000826. doi: 10.1371/journal.pdig.0000826 (PMC12011294; doi:10.1371/journal.pdig.0000826)
Supplement: Attachment — Submitted filename: Peer_Review_Response_PLOSDH_03_03_25.docx [file pdig.0000826.s008.docx]

We would like to thank the editor(s) and reviewers for their time and consideration of our manuscript. We have carefully reviewed our submission and have made some minor grammatical changes that were caught in the re-review. While our responses to Reviewer #4's comments remain substantively unchanged from our previous submission (dated 01-21-2025), we have now added clear references to the specific sections of the manuscript that were modified in response to their feedback, making these revisions more transparent and easier to track. These references are appended to the previous responses and are **bolded** + *italicized* to flag them. We welcome any additional discussion if Reviewer 4’s comments do not feel adequately addressed.

Reviewer #1: Thanks for engaging with the comments and providing a new version of the manuscript.

RESPONSE: We appreciate Reviewer #1’s comments and would like to thank them for their time and consideration in conducting the review. We feel that our manuscript has improved due to their input.

Reviewer #4: None of the feedback i provided was responded to or incorporated. You can find them here below

RESPONSE: We thank Reviewer #4 for their comment. We sincerely apologize for the apparent oversight. Upon reviewing our submission, we discovered that while we did incorporate your valuable feedback, we failed to clearly indicate where exactly these changes were made in our manuscript. In this submission, we have included ***line numbers and excerpts*** along with each response to flag our edits.

Thank you for inviting me to review this paper. This has been informative and educative for me. The manuscript generally offers new insights and new data, and it is clear that this area is under-research. I believe this paper will offer the required insights for others to follow suit. I must also add that the subject matter area (Artificial Intelligence) is not one that I am very familiar with but has limited knowledge in the area. This may affect my comment on this manuscript to an extent.

RESPONSE: We thank Reviewer #4 for taking the time to review our manuscript. We particularly appreciate their candid acknowledgment regarding familiarity with AI, as this supported our revisions to ensure the submission remains accessible to readers from various backgrounds.

Background
That being said, I am confident that the manuscript has a very good rigor to it. I was particularly interested in questioning the methodology (being that the qualitative approach is my research strength) but found that every decision was justified scientifically. However, my concern regarding the methodology is the richness of the responses provided by the study's virtual participants. I am saying this because speaking has been established to communicate feelings, attitudes, and perspectives more than informal writing. Personally, I also believe that people tend to exhibit laziness in responding to questions through writing (as long as they are not taking examinations).

RESPONSE: We agree that there are drawbacks to asynchronous online focus groups. While we address key limitations like moderation challenges and potential misunderstandings in our Limitations section, we have also strengthened our justification for this method in response to Reviewer #1 (comment 4). Our revised submission includes references to literature evaluating the richness of data and thematic content that asynchronous online focus groups can provide.

***See lines 116-121: There were also pragmatic reasons for selecting an asynchronous online variation. The format afforded patients greater flexibility and convenience to participate in the study and gave an extensive period to reflect on and contribute their responses compared to traditional focus groups [29]. Additionally, literature suggests that text-based focus groups generate similar thematic content and achieve similar data quality when compared to live variations, despite the overall amount of content being less [30].***

Method Section
While I agree that we can generate quantitative data in a qualitative paper, the authors still need to emphasize how or why they had to include multiple-choice questions. This, I believe was not clear in the paper.

RESPONSE: In accordance with similar feedback received from Reviewer #1 (comment 4), our revised submission includes additional justification for including multiple-choice questions and their relevance in combination with the qualitative data.

***See lines 122-127: Focus groups generated both qualitative and quantitative data through open-ended prompts and multiple-choice survey questions, respectively. Survey questions were included to support methodological triangulation, as well as to provide participants a structured starting point for some prompts [31]. This article focuses on the study’s qualitative findings, but also reports the survey data as a complement to the results. Our reporting follows guidance from the Standards for Reporting Qualitative Research (SRQR; see S1) [32].***

Result Section
Please shrink the excerpts to 1.0 line spacing in place of what is currently used. It helps to clearly distinguish the excerpts and the manuscript’s texts.

RESPONSE: We have reduced the line spacing for quotations.

***See lines 253-255 as one example.***

In several sub-themes, you have only included one excerpt. This, I believe may be limiting for your data (particularly because your participants are across the country). What validates the quality of qualitative research is identifying patterns in the participants’ responses, which is not reflected in some components of your study result. In other words, I find it more plausible if you can have a supplementary file where more excerpts per theme and/or sub-themes are included

RESPONSE: We thank Reviewer #4 for this thoughtful feedback on the presentation of our qualitative data. We have incorporated Reviewer #4’s suggestion and have included a supplemental table that identifies each information factor along with a corresponding participant quotation. We hope this provides additional support for the thematic without overburdening the narrative flow of the manuscript.

***See lines 243-244: Additional quotations highlighting each information factor are included in the supporting information section (see S3).***

Limitation Section
Kindly provide a heading for the Limitation section
I agree with all the limitations called out.

RESPONSE: Our revised submission includes a Limitations heading.

***See line 536: Limitations***

Conclusion
This reads well for me
